# The prehospital NEW score to assess septic shock in-hospital, 30-day and 90-day mortality

**DOI:** 10.1186/s12879-024-09104-7

**Published:** 2024-02-16

**Authors:** Romain Jouffroy, Florian Négrello, Jean Limery, Basile Gilbert, Stéphane Travers, Emmanuel Bloch-Laine, Patrick Ecollan, Josiane Boularan, Vincent Bounes, Benoit Vivien, Papa Gueye

**Affiliations:** 1 Intensive Care Unit, Ambroise Paré Hospital– Assistance Publique Hôpitaux Paris, 9 avenue Charles De Gaulle, 92100 Boulogne-Billancourt, Paris, France; 2 IRMES - Institute for Research in Medicine and Epidemiology of Sport, INSEP, Paris, France; 3 INSERM U-1018, Centre de recherche en Epidémiologie et Santé des Populations - U1018 INSERM, Paris Saclay University, Villejuif, France; 4 SAMU 972, Centre Hospitalier Universitaire de Martinique, Fort-de-France Martinique, France; 5 UR5_3 PC2E, University of the Antilles, French West Indies, France; 6 Department of Emergency Medicine, SAMU 31, University Hospital of Toulouse, Toulouse, France; 7 Paris Fire Brigade, Paris, France; 8 Emergency Department, Cochin Hospital, Paris, France; 9 Emergency Department, SMUR, Hôtel Dieu Hospital, Paris, France; 10 Intensive Care Unit, SMUR, Pitie Salpêtriere Hospital, 47 Boulevard de l’Hôpital, 75013 Paris, France; 11 Centre Hospitalier Intercommunal Castres-Mazamet, Castres, France; 12 Intensive Care Unit, Anaesthesiology, SAMU, Necker Enfants Malades Hospital, Assistance Publique - Hôpitaux de Paris, Paris, France

**Keywords:** Septic shock, Triage, Severity, Assessment, Prehospital setting, National early warning score 2

## Abstract

**Background:**

The early identification of sepsis presenting a high risk of deterioration is a daily challenge to optimise patient pathway. This is all the most crucial in the prehospital setting to optimize triage and admission into the appropriate unit: emergency department (ED) or intensive care unit (ICU). We report the association between the prehospital National Early Warning Score 2 (NEWS-2) and in-hospital, 30 and 90-day mortality of SS patients cared for in the pre-hospital setting by a mobile ICU (MICU).

**Methods:**

Septic shock (SS) patients cared for by a MICU between 2016, April 6th and 2021 December 31st were included in this retrospective cohort study. The NEWS-2 is based on 6 physiological variables (blood pressure, heart rate, respiratory rate, temperature, oxygen saturation prior oxygen supplementation, and level of consciousness) and ranges from 0 to 20. The Inverse Probability Treatment Weighting (IPTW) propensity method was applied to assess the association with in-hospital, 30 and 90-day mortality. A NEWS-2 ≥ 7 threshold was chosen for increased clinical deterioration risk definition and usefulness in clinical practice based on previous reports.

**Results:**

Data from 530 SS patients requiring MICU intervention in the pre-hospital setting were analysed. The mean age was 69 ± 15 years and presumed origin of sepsis was pulmonary (43%), digestive (25%) or urinary (17%) infection. In-hospital mortality rate was 33%, 30 and 90-day mortality were respectively 31% and 35%. A prehospital NEWS-2 ≥ 7 is associated with an increase in-hospital, 30 and 90-day mortality with respective RRa = 2.34 [1.39–3.95], 2.08 [1.33–3.25] and 2.22 [1.38–3.59]. Calibration statistic values for in-hospital mortality, 30-day and 90-day mortality were 0.54; 0.55 and 0.53 respectively.

**Conclusion:**

A prehospital NEWS-2 ≥ 7 is associated with an increase in in-hospital, 30 and 90-day mortality of septic shock patients cared for by a MICU in the prehospital setting. Prospective studies are needed to confirm the usefulness of NEWS-2 to improve the prehospital triage and orientation to the adequate facility of sepsis.

## Introduction

During the last twenty years, despite research on prevention and treatments, the mortality and the morbidity rates of sepsis remain stable [1, 2]. To date, whatever the initial stage of sepsis, this latter one is responsible of 20% of all deaths worldwide, approximately 11 million deaths ever year [3]. Sepsis is also a major cause of death among hospitalized patients, especially in intensive care unit (ICU) [4, 5] where mortality and morbidity rates are estimated of 37.3% and 10.4% [6].

On 2017 the World Health Assembly and the World Health Organization defined that prevention, diagnosis, and sepsis management are the main priorities to decrease morbidity and mortality [7]. It was underpinned that sepsis outcome depends on early identification and treatment implementation with hemodynamic optimization and antibiotic therapy (ABT) administration [8, 9] both included in a bundle of care [10].

In and, out-of-hospital sepsis diagnosis and severity evaluation are the bundle of care first steps aiming to determine the appropriate level of care. Because 70% of sepsis occurs outside a hospital environment, a special attention is needed to help physician for sepsis diagnosis and severity assessment to optimize triaging between ambulatory treatment, ward, emergency department (ED) or ICU admission [11]. Outside hospital setting, severity assessment is mainly based on clinical signs due to the absence of biomarker availability [12–14]. Different sepsis severity assessment scores have been developed, to enhance clinical diagnostic performance, despite to date no score was prospectively validated for pre-hospital use [15, 16].

In 2012, the Royal College of Physicians launched the National Early Warning Score (NEWS) to improve the outcomes of patients suffering from acute illness [17]. The NEWS score aims to triggering a rapid and effective clinical response, in time, person and place because the triad: early detection, timeliness of response and competency of the clinical response directly impact the outcome [18–25].

This study aims to describe the association between the prehospital National Early Warning Score 2 (NEWS-2) and in-hospital, 30 and 90-day mortality of SS patients cared for in the pre-hospital setting by a mobile ICU (MICU).

## Methods

### Population

As previously reported, in France, pre-hospital emergency system (PEMS) relies on the Service d’Aide Médicale d’Urgence (SAMU) [12, 13, 15, 26, 27]. Briefly, SAMU is composed of dispatch operators and emergency physicians [28] with a unique national phone number, the “15”. After a telephone discussion with the patient, or a relative, or a witness, the physician, based on patient’s medical history and reported symptoms, in case of life-threatening emergencies, may decide to dispatch a mobile intensive care unit (MICU) team to the scene. The MICU team, a driver, a nurse and an emergency physician, is equipped to face initial management of major organ failures [28].

All adults patients aged ≥ 18 years, cared for by a MICU from SAMU 75 Necker - Enfants Malades Hospital, SAMU 75 Lariboisière Hospital, SAMU 75 Pitié Salpêtrière Hospital, SAMU 75 Hôtel Dieu Hospital, Paris, Assistance Publique - Hôpitaux de Paris, Paris– France; Paris Fire Brigade Emergency Medical Service, Paris– France; SAMU 972 Fort de France University Hospital, La Martinique– France; SAMU 31 University Toulouse Hospital, Toulouse– France and SAMU 31 Castres Hospital, Castres– France, between 2016, April 6th, and 2021, December 31st, were included in this retrospective study based on 2012 sepsis-2 conference septic shock definition [29]. Eligible patients were identified using electronic research using septic shock or severe sepsis keywords. No exclusion criteria were used in this study.

### Ethical considerations

The French Society of Anaesthesiology and Intensive Care ethics committee (Reference: IRB00010254-2017-026, 2017/12/12), the Ethics Committee for Behavioural and Health Research (CERCES Reference 2018-04, 2018/01/16) and the National Heart Agency (2017-A02335-48–2017/07/30) approved the trial protocol waiving patient consent for this retrospective study.

### Data collection

A standardized data collection template was used in order to minimize data abstraction bias [30].

Patients’ demographic characteristics (age, weight, height, calculated body mass index (BMI) and gender), prehospital supposed origin of sepsis, initial prehospital vital signs values (systolic (SBP), diastolic (DBP) and mean blood (MBP) pressure, heart rate (HR), pulse oximetry (SpO2), respiratory rate (RR), body core temperature and Glasgow coma scale (GCS)), skin mottling score (SMS from 0 to 5), capillary refill time (CRT) (seconds), duration of prehospital care, prehospital treatments delivered (antibiotic therapy (ABT) type and dose, fluid volume expansion type and dose, catecholamine type and dose) were collected from MICU pre-hospital medical records. Comorbidities: hypertension, coronary heart disease (CHD), chronic cardiac failure (CCF), chronic renal failure (CRF), chronic obstructive pulmonary disease (COPD), diabetes mellitus and history of cancer, were also collected from pre and in-hospital medical reports. Length of stay in the ICU, length of stay in the hospital, 30 and 90-day mortality were retrieved from in-hospital medical records. In France, the hospital patient monitoring software enables to know the vital status even if the patient is no longer hospitalised. Thus, the vital status “alive” or “dead” on day-90 was available for each analysed patient.

Simplified acute physiology score (SAPS2) [31] was calculated 24 h after hospital admission.

### Statistical analysis

A mean with standard deviation was used to express quantitative parameters with a gaussian distribution, median with interquartile range [Q1-Q3] for quantitative parameters with a non-normal distribution and absolute values and percentages for qualitative parameters.

The main outcomes were in-hospital, 30 and 90-day mortality of septic shock patients initially cared for by a MICU in the pre-hospital setting.

The NEWS-2 was calculated based on the sum of the worst value of the 6 following physiological variables: blood pressure, heart rate, respiratory rate, temperature, oxygen saturation prior oxygen supplementation, and level of consciousness according to the 2017 Royal College of Physicians.

guidelines [17]. The NEWS-2 ranges from 0 to 20.

A NEWS-2 ≥ 7 threshold was chosen for increased clinical deterioration risk definition and usefulness in clinical practice based on previous report [17].

The relationship between each covariate and in-hospital, 30-day and 90-day mortality rates were assessed by bivariate and multivariate analyses. The NEWS-2 was analysed, as a continuous variable and as a binary variable using a threshold of NEWS ≥ 7 because its association with increased clinical deterioration risk. Results are expressed by an Odd Ratio (OR) and adjusted Odd Ratio (aOR) with a 95% confidence interval [95 CI].

A log binomial regression weighted with the inverse probability of treatment (IPTW) propensity score method was computed taking into potential cofounders. The propensity score aims to decrease bias due to non-randomized treatment allocation [32]. Cofounders included in the IPTW propensity analysis were: age, cancer history, CRF, COPD, CHD, diabetes mellitus, CCF, SAPS2, prehospital ABT administration, prehospital fluid volume expansion and prehospital catecholamine infusion. The selection of variables included in the multivariable analysis was done a priori guided by previous knowledge of factors known to influence septic shock survival. Results were expressed as adjusted risk ratio (RR) [95 CI].

All tests were 2-sided with a statistically significant *p-value* considered as < 0 0.05.

All analyses were performed using R 3.4.2 (http://www.R-project.org; the R Foundation for Statistical Computing, Vienna, Austria).

## Results

### Population characteristics

Between 2021, April 6th, and 2021, December 31st, 530 patients requiring pre-hospital MICU intervention for septic shock were analysed. The missing data rate was lower than 1%; these data were deleted for the statistical analysis.

Of the 7 participating centres, 165 patients (31%) were included by the Paris Fire Brigade Emergency Medical Service, 104 patients (20%) by SAMU 31 Toulouse, 77 patients (15%) by SAMU 75 Necker, 71 patients (13%) by SAMU Castres, 51 patients (10%) by SAMU 972 La Martinique, 31 patients (6%) by SAMU 75 Lariboisière, 14 patients (3%) by SAMU 75 Hôtel Dieu and 17 patients (3%) by SAMU 75 Pitié Salpétrière.

The overall population mean age was 69 ± 15 years and 341 patients (64%) were male gender.

One hundred eighty (34%) patients died during hospital stay, 164 (31%) patients had died by day-30, and 184 (35%) had died by day-90.

Table 1 summarise the populations’ demographic and clinical characteristics (Table 1).

**Table 1:** Population demographic and first prehospital clinical characteristics. Results are expressed as mean with standard deviation for quantitative parameters with gaussian distribution, as median with interquartile range for quantitative parameters with non-normal distribution and, as absolute value and percentage for qualitative parameters. *p*-value corresponds to the comparison between alive and deceased patients. *p*-value in bold indicates a significant difference between alive and deceased patients

	Overall population (*n* = 530)	In-hospital			On day 30			On day 90		
		Alive (*n* = 350)	Deceased (*n* = 180)	*P* value	Alive (*n* = 366)	Deceased (*n* = 164)	*P* value	Alive (*n* = 346)	Deceased (*n* = 184)	*P* value
***Demographics***										
Age (years)	69 ± 15	67 ± 15	73 ± 14	***< 10*** ^***− 3***^	68 ± 15	73 ± 14	***< 10*** ^***− 3***^	67 ± 15	74 ± 14	***< 10*** ^***− 3***^
Weight (kg)	74 ± 20	75 ± 20	70 ± 20	***0.026***	75 ± 20	70 ± 20	***0.014***	75 ± 20	71 ± 20	***0.038***
Height (cm)	170 ± 12	170 ± 13	169 ± 9	0.359	170 ± 13	169 ± 9	0.339	170 ± 13	169 ± 9	0.288
BMI (kg.cm^− 2^)	28 ± 3	29 ± 5	25 ± 6	0.075	29 ± 5	24 ± 6	***0.038***	29 ± 5	25 ± 6	0.140
Male gender	341 (64%)	231 (66%)	110 (61%)	0.111	243 (66%)	98 (60%)	0.141	224 (65%)	117 (64%)	0.179
***Comorbidities***										
Hypertension	230 (43%)	152 (43%)	78 (43%)	0.965	159 (43%)	71 (43%)	0.974	147 (42%)	83 (45%)	0.770
CHD	104 (20%)	60 (17%)	44 (24%)	***0.021***	64 (17%)	40 (24%)	0.065	59 (17%)	45 (24%)	***0.022***
CCF	134 (25%)	72 (21%)	62 (34%)	***< 10*** ^***− 3***^	74 (20%)	60 (37%)	***< 10*** ^***− 3***^	71 (21%)	63 (34%)	***< 10*** ^***− 3***^
Diabetes Mellitus	151 (28%)	108 (31%)	43 (24%)	0.162	109 (30%)	42 (26%)	0.326	107 (31%)	44 (24%)	0.102
COPD	79 (18%)	48 (14%)	31 (17%)	0.086	49 (13%)	30 (18%)	0.144	46 (13%)	33 (18%)	***0.059***
CRF	75 (14%)	42 (12%)	33 (18%)	***0.041***	45 (12%)	30 (18%)	0.069	39 (11%)	36 (20%)	***0.014***
Cancer history	186 (35%)	112 (32%)	74 (41%)	***0.017***	116 (32%)	70 (43%)	***0.015***	107 (31%)	79 (43%)	***0.005***
***Prehospital***										
First SBP (mmHg)	97 ± 30	98 ± 30	95 ± 31	0.245	99 ± 30	93 ± 30	0.055	98 ± 29	96 ± 31	0.445
First DBP (mmHg	58 ± 19	59 ± 19	56 ± 20	0.139	59 ± 19	55 ± 20	0.069	59 ± 19	57 ± 20	0.290
First MBP (mmHg)	71 ± 22	72 ± 22	69 ± 22	0.215	72 ± 22	68 ± 22	0.064	72 ± 22	70 ± 23	0.413
First HR (beats.min^− 1^)	114 ± 28	115 ± 27	112 ± 31	0.241	115 ± 28	113 ± 31	0.463	115 ± 27	112 ± 30	0.201
First CRF (sec)	4 ± 2	4 ± 3	4 ± 2	0.786	4 ± 2	4 ± 2	0.408	4 ± 3	4 ± 2	0.466
First RR (movements.min^− 1^)	30 [22–36]	28 [20–35]	32 [25–39]	***0.001***	28 [22–35]	31 [25–38]	***0.005***	28 [21–35]	31 [25–38]	***0.004***
First SMS	1 [0–3]	1 [0–3]	2 [0–3]	***0.005***	1 [0–3]	2 [0–4]	***0.022***	1 [0–3]	2 [0–4]	***0.004***
First SpO2 (%) **prior oxygen supplementation**	92 [85–96]	93 [87–96]	90 [84–95]	***0.014***	93 [87–96]	90 [83–95]	***0.006***	93 [86–96]	90 [84–95]	***0.047***
First body core temperature (°C)	38.5 [36.5–39.1]	38.5 [36.9–39.3]	38.0 [36.0–39.0]	***0.009***	38.4 [36.8–39.3]	38.1 [36.0–39.0]	***0.018***	38.5 [36.9–39.3]	38.0 [36.0–39.0]	***0.013***
First glycemia (mmol.l^− 1^)	8.7 [6.3–12.0]	8.8 [6.7–12.2]	7.9 [5.7–11.1]	***0.009***	8.9 [6.7–12.3]	7.9 [5.7–11.2]	***0.014***	8.9 [6.7–12.2]	7.9 [5.9–11.2]	***0.013***
GCS	15 [12–15]	15 [13–15]	14 [11–15]	***0.009***	15 [13–15]	14 [11–15]	***0.002***	15 [13–15]	14 [11–15]	***0.022***
First blood lactate level (mmol.l^− 1^)	5.8 ± 3.4	5.6 ± 3.4	6.3 ± 3.5	***0.025***	5.6 ± 3.3	6.3 ± 3.6	0.071	5.6 ± 3.4	6.3 ± 3.5	0.057
Fluid expansion (ml)	932 ± 573	954 ± 594	896 ± 547	0.061	944 ± 587	907 ± 542	0.522	949 ± 595	906 ± 547	0.432
Norepinephrine administration	155 (29%)	101 (29%)	54 (30%)	0.304	104 (28%)	51 (31%)	0.530	102 (29%)	53 (29%)	0.929
Norepinephrine dose	1.0 [0.5–2.0]	1.0 [0.5–2.0]	1.0 [0.8–2.0]	0.092	1.0 [0.5–2.0]	1.0 [1.0–2.0]	***0.041***	1.0 [0.5–2.0]	1.5 [1.0–2.0]	0.055
ABT administration	132 (25%)	97 (28%)	35 (19%)	0.089	97 (27%)	35 (21%)	0.205	88 (25%)	44 (24%)	0.529
Duration (min)	71 ± 34	60 ± 34	74 ± 35	0.123	69 ± 33	74 ± 35	0.111	70 ± 34	73 ± 34	0.242
NEWS-2	9 ± 3	9 [6–11]	10 [7–12]	***0.011***	9 [6–11]	10 [7–12]	***0.004***	9 [6–11]	10 [7–12]	***0.034***
NEWS-2 ≥ 7	406 (77%)	262 (75%)	144 (80%)	***0.037***	268 (73%)	138 (84%)	***0.007***	258 (75%)	148 (80%)	0.110
***In-hospital***										
SAPS2 score	60 ± 21	54 ± 19	71 ± 20	***< 10*** ^***− 3***^	54 ± 19	71 ± 21	***< 10*** ^***− 3***^	54 ± 19	70 ± 20	***< 10*** ^***− 3***^
ICU length of stay (days)	4 [2–8]	4 [2–8]	3 [1–8]	0.139	4 [2–9]	3 [1–7]	***0.007***	4 [2–8]	3 [1–8]	0.431
In-hospital length of stay (days)	10 [5–18]	12 [8–28]	5 [2–12]	***< 10*** ^***− 3***^	13 [8–21]	5 [2–11]	***< 10*** ^***− 3***^	12 [7–20]	5 [2–14]	***< 10*** ^***− 3***^

Legend: BMI = body mass index, SBP = systolic blood pressure, DBP = diastolic blood pressure, MBP = mean blood pressure, HR = heart rate, SpO2 = pulse oximetry, RR = respiratory rate, GCS = Glasgow coma scale, SMS = skin mottling score, CRT = capillary refill time, ABT = antibiotic therapy, CHD = coronary heart disease, CCF = chronic cardiac failure, CRF = chronic renal failure, COPD = chronic obstructive pulmonary disease, SAPS2 = Simplified acute physiology score, NEWS-2 = National Early Warning Score 2, ICU = intensive care unit

Presumed origin of septic shock was mainly pulmonary (43%), digestive (25%) or urinary (17%) (Table 2).

**Table 2:** Presumed origin of septic shock. Data are expressed as an absolute value with a percentage of all cases (due to rounding, total overpasses 100%)

Origin	n (percentage)
Pulmonary	230 (43%)
Digestive	130 (25%)
Urinary	88 (17%)
Cutaneous	33 (6%)
Meningeal	11 (2%)
Gynaecological	3 (0.5%)
Ear nose throat	2 (0.5%)
Cardiac	2 (0.5%)
Unknown	31 (6%)

### Prehospital setting

The mean duration of prehospital care was 71 ± 34 min, pre-hospital fluid expansion consisted on crystalloids (100%) with a mean volume of 932 ± 573 ml and 155 (29%) patients received norepinephrine infusion with a median dose of 1.0 [0.5–2.0] mg.h^− 1^.

Prehospital ABT was administered in 132 patients (25%) and no significant difference was observed between patients surviving or dying, at hospital, at 30 or 90 day (Table 1). The antibiotics were principally 3rd generation cephalosporins (*n* = 98, 75%).

### Hospital stays

The median length of stay in the ICU was 4 [2–8] days and the median in-hospital length of stay was 10 [5–18] days.

The mean SAPS2 score was 60 ± 21 with significant difference between patients surviving or dying in hospital, at day-30 and day-90 (Table 1).

### Prehospital NEWS-2

The mean overall prehospital NEWS-2 was 9 ± 3, with 406 patients (77%) patients having a prehospital NEWS-2 ≥ 7.

Bivariate logistic analysis revealed a significant association between prehospital NEWS (continuous variable) and in-hospital mortality (OR = 2.14 [1.19–3.83], *p* = 0.01), 28-day mortality (OR = 2.41 [1.33–4.36], *p* = 0.003) and 90-day mortality (OR = 1.88 [1.05–3.35], *p* = 0.03).

Using a threshold of a prehospital NEWS-2 ≥ 7, the association remains significant for in-hospital mortality (OR = 1.62 [1.04–2.58], *p* = 0.04), 28-day mortality (OR = 1.94 [1.22–3.18], *p* = 0.01) but not for 90-day mortality (OR = 1.43 [0.93–2.24], *p* = 0.11).

### Propensity IPTW analyses

The log binomial regression weighted with the IPTW observed an association between a prehospital NEWS-2 ≥ 7 and in-hospital mortality: RRa = 2.34 [1.39–3.95], 30-day mortality: RR = 2.08 [1.33–3.25] and 90-day mortality: RRa = 2.22 [1.38–3.59].

Initial lactate was missing for 218 patients (41%) and initial skin mottling score missing for 220 patients (42%); both covariables were not included in the model.

The calibration statistic values were: 0.54; 0.55 and 0.53 respectively for in-hospital mortality, 30-day mortality and 90-day mortality.

## Discussion

Here we report a positive association between in-hospital, 30 and 90-day mortality and (i) the prehospital NEWS-2, (ii) prehospital NEWS-2 ≥ 7 and among septic shock patients cared for in the pre-hospital setting by a mobile intensive care unit.

Previous reports underpinned that around one-third of potentially preventable deaths in the United Kingdom were related to poor clinical monitoring and/or inadequate response to clinical deterioration supporting that patients should be addressed to the most appropriate setting for clinical care [33], justifying NEWS-2 widespread deployment [17]. It is all the most true for sepsis for which diagnosis, severity assessment and treatment initiation does not suffer from any delay [14, 34, 35]. Beyond out- and in-hospital care aiming to improve sepsis outcome, early diagnosis and severity are cornerstones to decrease sepsis related mortality by initiation of the sepsis survival chain [36]. Early diagnosis is also recognised as an essential leverage arm to prevent potentially preventable deaths as it allows early initiation of treatment. Daily, PEMS are faced to a challenge aiming to, as quickly as possible, establish a right diagnosis and assess severity to adequately guide the patient towards the optimal care pathway for his or her disease. To establish a diagnosis and a clinical monitoring, simple and objective clinical tools usable at any time and reproduced by different caregivers are necessary. This is all the more important when diagnostic certainty is uncertain and/or urgent, even when combining both clinical and paraclinical, for example biological, variables, in the grey zone decision making [37]. Some clinical parameters, e.g., SMS, CRT [12, 13], are subjective and may be caught out in some situation, for example hypothermia.

Because of the lack of specificity of a single clinical sign [14], scoring system was developed to improve sensitivity and sensibility. For sepsis, the most known scores are which SOFA [38], Mortality in Emergency Department Sepsis (MEDS) [39], Predisposition, Infection, Response and Organ dysfunction (PIRO) [40] and q-SOFA since 2016 [14]. Although the latter does not require biological results and is recommended because of its simplicity outside ICU [41], q-SOFA validity remains under debate [13, 15, 16, 40, 42–44]. To date, in the prehospital setting, no score is validated, thus, sepsis severity assessment and prognostication still remain on clinical evaluation [45].

The score has several advantages, including: its ease of establishment since all the variables are accessible in the pre-hospital setting, its inter-observer reproducibility, and the possibility of being repeated over time in order to evaluate the treatment effect. However, one of the weakness is that NEWS-2 does not include age and major comorbidities, both reflecting frailty [46, 47] and associated with poor sepsis outcome [48–50].

### Limitations

The current study suffers from limitations. Because of the retrospective study design, we cannot exclude that a potential selection bias affects the results validity. In addition, we are unable to conclude on a causal link between the NEWS-2 and mortality related to septic shock and sepsis. We cannot exclude unknown or missed confounders during the analysis. The NEWS-2 performance and external validation need to be confirmed by prospective studies, although the inclusion of centres of varying size and geography (a large city - Paris, one medium-sized city - Toulouse and one rural city– Castres) seems promising and represents a study strength. The study population was only adults; consequently, results extrapolation to sepsis and to paediatric population is not possible. We should keep in mind that some NEWS-2 variables may be influenced by patient previous medications, e.g., beta-blocker therapy, restricting their contribution to the NEWS-2.

Beyond all these limitations, the NEWS-2 seems to be an adequate tool for pre-hospital sepsis screening of a high risk of poor evolution and should be considered as an aid to clinical decision making, not a barrier or alternative to skilled clinical judgement.

## Conclusion

Among sepsis patient requiring prehospital mobile intensive care unit intervention, a prehospital NEWS-2 ≥ 7 is associated with an increase in in-hospital, 30 and 90-day mortality. However, prospective studies are needed to confirm the usefulness of NEWS-2 to improve the prehospital triage, patient orientation to the optimal pathway and sepsis related mortality.

## Data Availability

The dataset analyzed during the current study are not publicly available because their containing information that could compromise the privacy of *research* participants but are available from the corresponding author on reasonable request.

## Abbreviations

BMI: Body mass index
MICU: Mobile intensive care unit
aHR: Adjusted hazard ratio
ED: Emergency department
ICU: Intensive care unit
SAMU: Urgent Medical Aid Service
ABT: Antibiotic therapy
SBP: Systolic blood pressure
DBP: Diastolic blood pressure
MBP: Mean blood pressure
HR: Heart rate
SpO2: Pulse oximetry
RR: Respiratory rate
GCS: Glasgow coma scale
SMS: Skin mottling score
CRT: Capillary refill time
SOFA: Sequential Organ Failure Assessment
qSOFA: Quick Sequential Organ Failure Assessment
SAPS-2: Simplified Acute Physiology Score
PEMS: Prehospital emergency medical service
RRa: Adjusted risk ratio
ORa: Adjusted odd ratio
CHD: Coronary heart disease
CCF: Chronic cardiac failure
CRF: Chronic renal failure
COPD: Chronic obstructive pulmonary disease
IPTW: Inverse Probability Treatment Weighting
NEWS-2: National Early Warning Score 2
